# Airborne Algae and Cyanobacteria Originating from Lakes: Formation Mechanisms, Influencing Factors, and Potential Health Risks

**DOI:** 10.3390/microorganisms13071702

**Published:** 2025-07-20

**Authors:** Xiaoming Liu, Tingfu Li, Yuqi Qiu, Changliang Nie, Xiaoling Nie, Xueyun Geng

**Affiliations:** 1 Shandong Provincial University Laboratory for Protected Horticulture, Weifang University of Science and Technology, Weifang 262700, China; 2 School of Environment and Geography, Qingdao University, Qingdao 266071, China; 3 Environmental Technology, School of Industrial Technology, Universiti Saints Malaysia, Pulau Pinang 11800, Malaysia; 4 School of Water Conservancy and Environment, University of Jinan, Jinan 250022, China; 5 State Key Laboratory of Microbial Technology, Shandong University, Qingdao 266237, China

**Keywords:** airborne algae, human health, lake spray aerosol, cyanobacteria

## Abstract

Algal and cyanobacterial blooms are anticipated to increase in frequency, duration, and geographic extent as a result of environmental changes, including climate warming, elevated nutrient concentrations, and increased runoff in both marine and freshwater ecosystems. The eutrophication of aquatic environments represents a substantial threat to human health. As eutrophication progresses, airborne algae and cyanobacteria, particularly harmful genera originating from aquatic environments, are released into the atmosphere and may pose potential risks to human health. Furthermore, respiratory distress has been documented in individuals exposed to aerosols containing harmful algal bloom (HAB) toxins. This review investigates the generation of aerosolised harmful algal blooms, their responses to environmental factors, and their associated health risks. Evidence suggests that airborne algae, cyanobacteria, and their toxins are widespread. When these are aerosolised into micrometre-sized particles, they become susceptible to atmospheric processing, which may degrade the HAB toxins and produce byproducts with differing potencies compared to the parent compounds. Inhalation of aerosolised HAB toxins, especially when combined with co-morbid factors such as exposure to air pollutants, could present a significant health risk to a considerable proportion of the global population. A more comprehensive understanding of the chemical transformations of these toxins and the composition of harmful algal and cyanobacterial communities can improve public safety.

## 1. Introduction

The phytoplankton community typically comprises cyanobacteria and various eukaryotic algal species [1]. Harmful algal blooms (HABs) have been documented in both marine and freshwater ecosystems. Both freshwater and marine aquatic phytoplankton blooms carry significant global implications for public health and ecosystem services [1,2]. Adverse effects stem from high bloom intensity or the presence of toxin-producing phytoplankton species [1]. While contemporary research predominantly focuses on aquatic environments, airborne algae have received considerably less attention compared to their aquatic counterparts [2]. Bioaerosols are airborne particles of biological origin, which include microorganisms and their byproducts [3]. As a type of bioaerosol, airborne algae play a crucial role in influencing global climate change and directly or indirectly impacting human health [2,3,4]. However, our understanding of the behaviour of airborne algae remains limited, owing to the few research studies conducted. Algae aerosols are primarily generated through processes such as wave breaking and bubble bursting [2]. The composition of airborne algal species is largely determined by the diversity of aquatic species. This underscores the importance of investigating how harmful algae in aquatic environments influence the distribution of airborne algae [2]. The frequency of aerosolised harmful algae is increasing, suggesting that rising temperatures facilitate the intensification of HABs and potentially other types, thereby expanding the threat to human health [5,6].

Recent research has increasingly focused on the presence of harmful algal toxins in the airway. Under specific environmental conditions, toxins produced by HABs can become aerosolised, leading to inhalation and subsequently causing adverse health effects [7]. The public health burden associated with aerosolised HABs and their toxins is anticipated to be significant. Some studies have demonstrated a marked increase in liver disease incidence among residents near eutrophic lakes, such as Lake Erie and Lake Taihu [8,9]. Another study identified common algal or cyanobacterial toxins in freshwater environments, including microcystins (MCs), anatoxins (ATXs), and cylindrospermopsin (CYN) [10]. Due to global warming, the frequency and intensity of cyanobacterial HABs in freshwater systems are rising globally, posing a threat to human health [11]. Previous studies have predominantly concentrated on the health impacts of individual HAB species or provided limited reviews of the diverse symptoms linked to various exposure routes of HABs [7]. However, the effects of these toxins on human airways remain underexplored.

After the experience of the COVID-19 pandemic, the potential adverse effects of bioaerosols on human health have garnered widespread attention. In specific environments, airborne algae and cyanobacteria constitute a significant portion of the atmospheric biological load, traditionally dominated by bacteria and fungi [12]. Research on bioaerosols and microorganisms in particulate matter has expanded considerably in recent years. However, substantial gaps remain in our understanding of aerosolised harmful algae and the toxins they produce. Therefore, this study conducts a comprehensive review of contemporary relevant literature to systematically explore the aerosolisation, transport, and inhalation processes of freshwater HAB toxins, as well as to provide an in-depth assessment of current methodologies for evaluating human exposure to aerosolised HAB toxins. Additionally, this study synthesises existing knowledge in the field, offering new insights and perspectives for advancing the understanding of airborne algae and their associated toxins.

## 2. The Formation and Environmental Factors on Airborne Algae and Their Toxins

### 2.1. The Aerosolised Algae Produced from the Aquatic Environment

**Figure 1:**
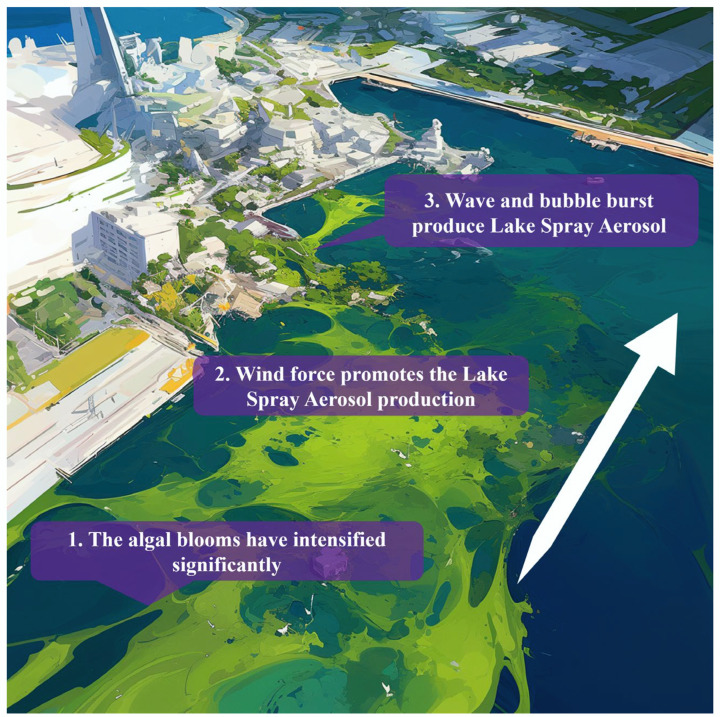
A scheme of airborne algae, cyanobacteria and their toxin production.

The primary mechanism of aerosolisation from blooms occurs through the generation of aerosols in freshwater bodies (lake spray aerosols; LSAs), as shown in Figure 1. LSAs are produced by freshwater wave breaking and bubble bursting [11]. There are other potential sources of aerosolisation; for example, underwater gas emissions through biogeochemical reactions and physical disturbances during recreational activities (boating, swimming, and splashing) can also lead to bubble bursting and aerosol formation [2]. Another point of aerosols produced by their HABs is the mixture of biota (bacteria, cyanobacteria, viruses, fungi, etc.) [13,14]. When wind or wave motion causes bubbles to form on the surface of a body of water, algal cells, attached microorganisms, and dissolved toxins become encapsulated in the bubbles, and at the moment the bubbles burst, droplets containing microorganisms, toxins, etc., are ejected into the air, forming aerosols [2]. Mixed microorganisms may increase toxicity and pose a greater threat to humans. HAB toxins may be released. These toxins become aerosolised as breaking lake waves eject jet and film droplets from bursting surface bubbles, forming LSA [12]. Lake spray aerosol particles have a bimodal size distribution, mainly fine particles, and may contain ultrafine particles [5,11,15].

### 2.2. Long-Range Transport of Airborne Algae

Long-range transport of LSA is dominated by atmospheric circulation (e.g., prevailing winds). Studies have shown that LSA can spread inland from the surface of lakes through the movement of air masses. For example, in studies in northern Michigan, USA, LSA was transported by winds from Lake Michigan to inland stations 25 km away and long-range transport of airborne algae cause potential threats [16,17]. Wood et al. detected microcystin-containing aerosols at a freshwater site 20 m from the shore at a height of 30 m, suggesting that freshwater aerosols can propagate overhead and also inland [18]. During cyanobacteria HAB events, microcystin can be transported for many kilometres in atmospheric particles without degradation due to its exceptional stability [2]. In addition, an analysis of LSA generated from water samples from different locations in Lake Michigan and Lake Erie revealed that its chemical composition reflects that of freshwater and that organic and biogenic substances increase with increasing cyanobacterial content, implying that the chemical composition of LSA changes during horizontal transport and that it may carry substances such as algal toxins [19].

### 2.3. The Concentration and Species of Algae and Cyanobacteria Determined the Airborne Distribution

**Figure 2:**
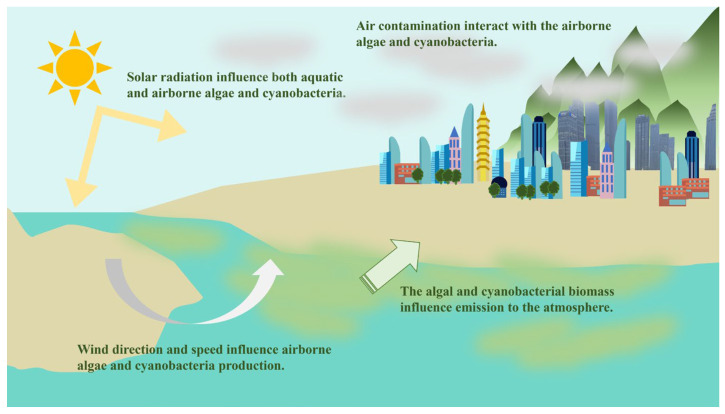
A summary of factors that influence the airborne algae and cyanobacteria.

Many factors can influence aerosolised algae and their toxins’ production, distribution, and transportation as shown in Figure 2. Due to the presence of airborne microorganisms originating from various sources, the air in areas close to aquatic environments is significantly influenced by the local microbial community [20]. The main contributing factors include algal concentration and species diversity of algae, which collectively shape the composition of airborne algae and their associated toxins. During algae bloom, elevated biomass concentrations lead to an increased proportion of biological material in the suspended particulate matter [17]. Toxins are typically not released through cell lysis caused by shear stress or turbulence, but rather they are released during cellular senescence, exposure to elevated salinity, or as a result of viral-induced lysis [2].

The differences in algal bloom states (severe algal blooms in Lake Mona and less-algal blooms in Lake Muskegon) lead to differences in the distribution of microcystins in aerosols, and particle size plays a key role in this [11]. A study revealed that bioaerosols, including airborne microalgae and cyanobacteria, may become fragmented while suspended in the air, resulting in smaller particles, or they may be emitted directly as bioparticle fragments [21]. Furthermore, microalgae and cyanobacteria were predominantly found in particles with a diameter exceeding 3.3 μm over the sea, but smaller particles are more likely to be transported further inland by wind [22].

### 2.4. Meteorological and Environmental Factors Influence the Airborne Algae, Cyanobacteria, and Toxin

Many environmental factors can influence the airborne algae and their toxins. For instance, although contemporary work has not determined the effects of temperature variation on airborne algal toxins, global warming accelerates eutrophication [7]. Recurrent and widespread intensification of lake phytoplankton blooms has been observed globally since the 1980s [1]. Humidity levels can influence the size and induce phase separation of aerosol particles. Low-humidity environments may favour the production and release of toxins by some algae, while high-humidity environments may facilitate the deposition and removal of toxins [23,24,25]. Additionally, low humidity can lead to particle shrinkage, increasing inhalation risk [24].

Wind speeds, patterns, and storm events significantly influence the quantity and dispersion of aerosol particles, potentially impacting inland regions [25]. Additionally, local wind patterns can affect toxins’ concentration and exposure levels in coastal and inland areas, even those distant from the original algal bloom site [24]. Wind speed also affects the production and transmission of LSA and airborne algae [26,27]. After LSA discharge, most particles settle near the lake, especially at low wind speeds, where dispersion is limited to the shoreline [26]. In contrast, under strong wind conditions, LSA can be lifted to high altitudes and transported with air currents more than 1000 km inland [26]. The seasonal cycle also influenced the distribution of airborne algae. Abundant airborne algae and cyanobacteria were more commonly observed during warmer seasons. However, the occurrence of specific genera varied depending on the month [25].

### 2.5. Airborne Chemical Compositions Interact with Airborne Algae and Toxins

Like temperature, chemical compounds, such as nitrogen, phosphorus, and organic composition, play an important role in facilitating eutrophication and subsequently influence the aerosolised HABs. Additionally, unlike the aquatic environment, the atmospheric condition creates specific conditions that will influence the chemical reaction with the airborne algal toxins. In aerosols, unique conditions, including elevated oxidant concentrations at the air–liquid interface (ALI), enhanced uptake of hydrophobic gases, and localised pH variations between the interface and the interior, may facilitate the transformation of toxins [24]. Such transformation may produce byproducts with varying potencies compared to the initial toxins. HAB toxins are susceptible to modification through multiple mechanisms similar to those experienced by airborne VOCs. Reactions in aerosols can proceed through (1) interfacial heterogeneous and bulk reactions involving a variety of gaseous oxidants, (2) photo-catalysed reactions occurring both at the core and interface, and (3) photochemical reactions, predominantly at the interface [24].

## 3. The Current Sample Collection and Detection Methods

### 3.1. Sample Collection and Analysis

**Figure 3:**
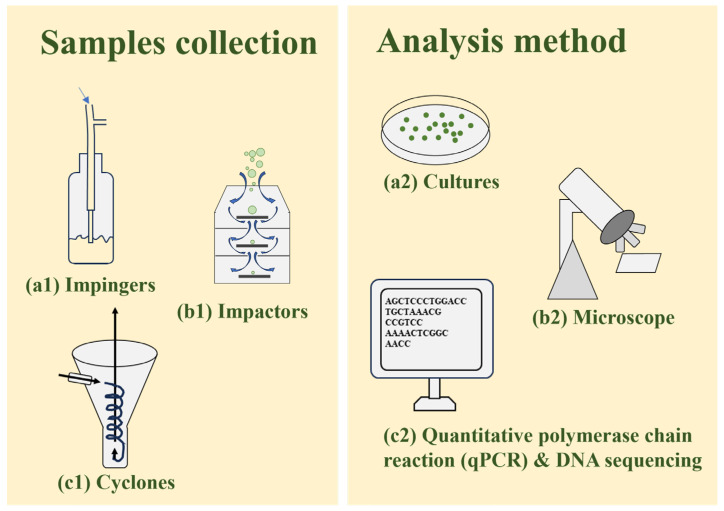
Several sample collection and analysis methods for airborne algae and cyanobacteria.

The prevailing aerosol sampling technique involves utilising a filter sampler, cascade impactor, or impinger as shown in Figure 3. Filter samplers draw airflow through a filter medium (e.g., quartz fibre), effectively capturing most aerosols below the size threshold set by a pre-selector device (typically a cyclone with PM_2.5_ or PM_10_ cut-off) [28]. Samplers require a pump or external power source to draw air at a specified flow rate and collect airborne particles onto a collection medium. Common particle collection mechanisms include interception, impaction, diffusion, and electrostatic attraction [28].

Cultivation is also a widely used method to analyse the airborne algae. Preparing the medium on which the bioaerosols will be taken is the first step of the research. Microorganisms respond differently to stress; therefore, choosing the right medium to culture will determine the survival of the airborne organism [25]. Several media were used, including sterile Bold basal medium, sterile mineral f/2 culture medium, and agarised medium [25]. It is important to note that high-volume airflow imposes significant stress on cyanobacteria and microalgae. Consequently, samplers typically feature low air flows, around 30 L/min. Examples include the Andersen cascade impactor sampler, which selectively collects particles based on size, ranging from less than 1.1 μm to over 7 μm. As air moves through successive stages of the impactor, its velocity gradually increases, allowing for the collection of smaller particles on Petri dishes positioned at each stage. Due to their inertia, these particles impact and adhere to the agar surface [25]. After the cultivation, the samples will be examined under a light microscope [25].

Another essential technique is the rapid advancement of high-throughput sequencing technology [28]. Advanced equipment now enables the detection of microbial compositions in micro-volume samples [28]. Contemporary studies have evaluated microbial communities associated with particulate matter across diverse sampling sites, including mountains, urban areas, rural regions, and specific locations [29,30]. While many studies focus on bacteria and fungi, it should be noted that cyanobacteria are often classified under bacteria. Given the complex characteristics of cyanobacteria and algae, we argue that the significance of their airborne nature deserves attention.

Given that most research regarding microorganisms in airborne particulate matter has focused on the analysis of cyanobacteria, this review primarily compares studies that also targeted cyanobacteria (Table 1). For example, in urban areas of Shanghai, cyanobacteria adhered to PM_2.5_ and accounted for 8.53%, making them the second most abundant genus in the microbial community [31]. Another study investigated the bacterial community structure that adheres to PM_2.5_ during the heating period in winter in Jinan and found that cyanobacteria account for 15.95% as the third abundant phylum [32]. Similarly, abundant cyanobacteria were found in PM_10_ samples in southeastern Italy, especially in some samples where they accounted for over 80% [33]. The bacterial community structures were compared between daytime and nighttime in Hangzhou. They also detected a high relative abundance of cyanobacteria in their samples; in particular, the relative abundance of cyanobacteria in the sample collected on 4/16/2015 was approximately 100% [34]. A similar phenomenon was found in Seoul. The cyanobacteria varied and dominated in the 1 W sample group (sunny week) [35]. The bacterial community structure were compared in TSP, PM_10_, and PM_2.5_ on different AQI value days, and the result revealed that the average relative abundance of cyanobacteria reached 18% as the second abundant phylum [36]. Simultaneously, they found cyanobacteria exhibited the lowest relative abundance of 10.25 ± 0.10% in the PM_10_ samples, whose relative abundance was 0.58 times lower than that in the PM_2.5_ samples (24.32 ± 0.31%, *p* < 0.005) and 0.52 times lower than that in the TSP samples (21.41 ± 0.36%, *p* < 0.05) [36]. The contemporary result revealed that cyanobacteria are a universal species existing in the ambient air [25]. Unfortunately, few studies have been conducted to determine the fate and potential risk. Future studies should focus on the variation of airborne cyanobacteria in response to specific environmental factors and their potential health risks.

In addition, significant advancements in satellite remote sensing have facilitated the detection and management of HABs by environmental managers and government agencies [38,39]. Using satellite remote sensing enables the acquisition of spatiotemporal coverage that surpasses the capabilities of conventional instruments and surveys [40]. Remote sensing can exploit specific optical signals associated with HABs, such as chlorophyll-a and other diagnostic photopigments, to develop algorithms for HAB detection [7]. As discussed above, we propose that a synthesised approach should be adopted. For example, satellite remote sensing can monitor algal blooms at a large scale. Once the advent of airborne algae occurs, complementary methods can be applied. The analysis would benefit from integrating HTS technology with cultivation techniques. Although cultivation can only identify a limited number of genera, it provides valuable information regarding viability. Additionally, HTS technology enables the identification of algal communities and offers precise data on specific airborne algae. Cultivation methods—including medium composition, growth conditions, and other parameters—can also be employed to enhance the overall assessment. Integrated culture-based and metagenomic profiling of airborne microbes holds promise for improving a more integrated perspective [41].

### 3.2. The Algal and Cyanobacterial Toxins Analysis and Potential Human Risk Assessment

Similar to detecting algal toxins in aquatic environments, airborne samples can be quantified in terms of toxins. Some research proposed that toxin detection was employed using liquid chromatography coupled with tandem mass spectrometry (LC/MS/MS) [7]. For example, Shi et al. analysed toxins in collected aerosol samples using LC/MS/MS [42]. The use of LC/MS/MS can also identify and quantify specific toxin congeners. Nicole E. Olson et al. measured 12 microcystin congeners using liquid chromatography triple quadrupole mass spectrometry (LC-MS/MS) [11]. ELISA kits are available for the detection of specific algal toxins, including brevetoxin, okadaic acids, and others [43].

Regarding the potential risk to human health, it is well known that the suspended microorganism can enter the human respiratory tract through respiration and may cause allergic reactions or infections [44]. In addition, certain biological factors may trigger respiratory allergic reactions in humans through airborne pathways [45]. Biological constituents released during harmful algal blooms (HABs), such as cyanobacterial toxins, bacterial endotoxins, and algal cellular debris, may synergistically enhance toxicity. For example, co-exposure to cyanotoxin and endotoxin may exacerbate respiratory inflammation [7]. Research revealed that an adult human inhaled approximately 240 algal cells/h in a tidal breathing zone [45]. Heise et al. correlated airborne algae with symptoms of hay fever [46]. The allergenic potencies of *Phormidium fragile* and *Nostoc muscorum*, two frequent viable algae in the atmosphere of Varanasi City, India, were evaluated using an intradermal allergy test and subsequent leukocyte counts. They found that both species varied in their allergenic potency. *N*. *muscorum* appeared to be more allergenic than *P*. *fragile*. However, when the allergens were mixed in equal amounts, these varieties of allergenicity increased significantly. A limited cross-reactivity pattern between the species was also evident [12].

As for the algal toxins, they also pose risks to health [47]. For instance, an evaluation of health using an animal model conducted to estimate the effects of airborne aerosolised algal toxins on Drosophila. The results demonstrated that a single exposure to aerosolised harmful algal bloom (HAB) particles poses significant immediate and long-term health risks [47]. Notably, the age at the time of exposure plays a critical role in determining the specific nature of the impact caused by aerosolised HABs. In addition, laboratory studies have shown that mice are 10 times more sensitive to inhaled microcystins than to oral ingestion. After inhaling microcystins, mice experience microscopic damage to their nasal passages and livers, resulting in systemic effects [11]. This raises concerns about unintended exposure for people living near or downwind of HABs, professionals exposed to HABs, and those engaged in recreational activities in HAB-affected freshwater environments [11]. A tolerable daily intake (TDI) for intranasal inhalation of MC-LR in humans was calculated based on animal studies that assessed the toxicity of MC-LR in mice and considered human body parameters. Using a daily air inhalation volume of 7 m^3^/day and a body weight of 60 kg, the derived TDI for MC-LR via intranasal exposure is 17 ng/m^3^/day [48].

Moreover, more people have realised the potential risk of aerosolised harmful algal toxins and have assessed their adverse effects on human organs and even cellular structures. For instance, the impact of aerosolised MC-LR exposure on a 3D human airway epithelium model derived from 14 healthy donors was investigated. This 3D model, cultured at an air–liquid interface, consists of multiple cell layers with functional cilia and mucus production. The study assessed the effects of MC-LR on cell viability, mucociliary clearance, tissue integrity, and cilia beat frequency [49].

## 4. The Airborne Algal and Cyanobacterial Toxin Risk on Human Health

### 4.1. The Toxic Mechanism on Human Health

**Figure 4:**
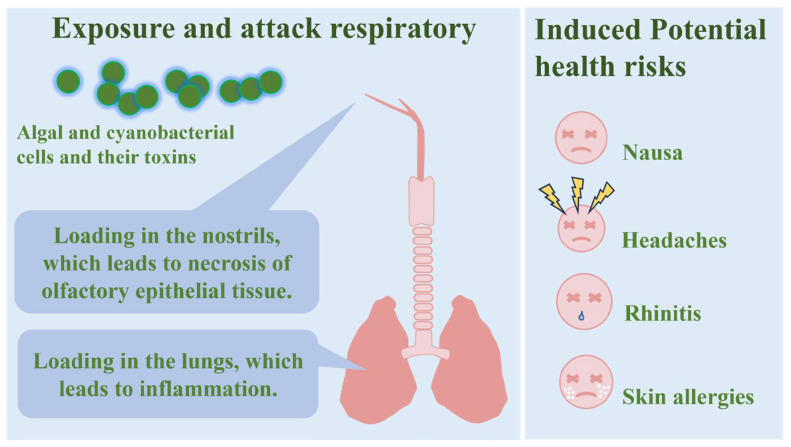
Potential human health risk exposure to the airborne algae, cyanobacteria, and their toxins.

As mentioned in the introduction, we discussed the potential risks of aerosolised algal toxins on human health as shown in Figure 4. Microcystins produced by cyanobacteria (e.g., *Microcystis* spp.) disrupt cellular signalling pathways by inhibiting protein phosphatases, leading to the apoptosis or necrosis of hepatocytes, and may induce hepatocellular carcinoma with long-term exposure [25]. Another study exposed a fully differentiated three-dimensional (3D) human airway epithelium to an MC-LR-containing aerosol once daily for three consecutive days. The concentrations of MC-LR ranged from 100 pM to 1 µM. Several inflammation-related genes were found to be upregulated, including C-C motif chemokine 5 (CCL5; log2FC = 0.57, *p* = 0.03) and C-C chemokine receptor type 7 (CCR7; log2FC = 0.84, *p* = 0.03). Functionally, conditioned media collected from the MC-LR-exposed airway epithelium exhibited significant chemoattractive properties for primary human neutrophils. Moreover, the levels of secreted chemokine proteins in the conditioned media were elevated, such as CCL1 (log2FC = 5.07, *p* = 0.0001) and CCL5 (log2FC = 1.02, *p* = 0.046). These findings suggested that the exposure of the human airway epithelium to MC-LR can induce an inflammatory response, which may contribute to the development of acute or chronic respiratory diseases [49]. It would be valuable to explore not only MC-LR but also other types of algal toxins and their effects.

An investigation into the levels of MC in the nasal mucosa of participants during a cyanobacterial bloom caused by *Microcystis aeruginosa* revealed that 95% exhibited concentrations of MC above the limit of detection, with significantly higher MC concentrations observed among individuals who had direct exposure to affected waters compared to those without recent contact [50]. Secondly, phytoplankton, as one of the bioaerosols, exhibits the capability to absorb and adsorb polycyclic aromatic hydrocarbons (PAHs), which are toxic pollutants in marine and atmospheric environments. The environmentally significant fraction of PAHs that is bioavailable can accumulate in organisms. Since bioaccumulation by phytoplankton often represents the initial step for chemicals entering the aquatic food web, toxic substances accumulated in the aquatic environment may be transported into the atmosphere via bioaerosols, subsequently creating a risk for the human respiratory tract [25].

### 4.2. Aerosolised HABs Events

The risks to human health posed by algal toxins have been reported. For example, recreational activities involving high levels of total cyanobacteria exposure were found to be significantly associated with an increased prevalence of low-severity respiratory symptoms, and results showed that the high-exposure group had a 2.1-fold higher rate of respiratory symptom reporting compared to the low-exposure group, with symptoms primarily mild (e.g., cough, sore throat) [51] These events suggest the harmful algae and cyanobacteria can affect the respiratory system. Therefore, safeguarding public health requires increased attention.

## 5. Future Perspective

As discussed, aerosolised harmful algal blooms exist ubiquitously, and the potential risk can be species-dependent. Contemporary references are limited in understanding the profound effects. One of the limitations of aerosolised HAB research is the insufficient direct observation of airborne HABs. Previous bioaerosol research has included analysis of cyanobacteria [32,34], albeit with limited depth. Moreover, even with the application of high-throughput sequencing technology, the more precise identification of airborne algae at the species level is recommended, as many current results remain categorised under generic labels such as ‘unclassified’ or ‘uncultured’. Furthermore, during harmful algal bloom aerosol sampling campaigns, it is crucial to monitor various meteorological conditions. Elevated wind speeds, extensive fetches, photosynthetically active radiation (PAR), and relative humidity (RH) can significantly influence cyanotoxin aerosol concentrations, affecting the levels of airborne cyanotoxins that reach human populations. Our current understanding of the production of aerosolised algal toxins and their implications for air quality remains limited. Therefore, it is recommended that future studies strengthen the relevant analyses.

Additionally, the ostensibly benign home environment harbours a variety of hazardous substances emitted from domestic activities, posing significant risks to human health. This concern is particularly pronounced given that individuals spend approximately 80% of their time indoors. In the context of indoor air quality, both at home and in work environments, attention is frequently directed towards microbes, primarily bacteria and fungi. However, airborne microalgae and cyanobacteria within indoor settings remain under-investigated. The air conditioning (AC) filters are also promising samples for research on microbiota and HABs. For example, a research studied microbiota on AC in Shanghai using high-throughput sequencing technology based on ITS and 16S rRNA gene, unveiling the contamination caused by bacteria, fungi, and pollen [52]. In another study, a culture-independent approach was employed, combining qPCR and high-throughput sequencing technologies to investigate the community structure of cyanobacteria. The findings revealed that cyanobacterial concentrations exhibited seasonal variations, with the highest levels observed during autumn, recurring periodically and approximately 2.60- and 10.57-fold higher than winter and summer, respectively [53]. Another recent study also detected cyanobacteria in indoor environments and revealed that cyanobacteria constituted 29.0% of the bacterial community at the remote controller, making it the most dominant phylum at this location [41]. Furthermore, certain cyanobacteria have been identified in terrestrial environments, including airborne cyanobacteria at agricultural sites [54]. Future research should also address the potential respiratory health risks related to the soil environment.

## 6. Conclusions

HABs are increasing in frequency and geographic distribution due to warming temperatures and various environmental factors, posing a substantial threat to public health. Inhalation of aerosolised HAB toxins has been associated with respiratory distress; however, the potential transformation of these toxins through atmospheric processes, which could produce more potent derivatives, remains poorly understood. This study underscores the necessity of investigating the combined impacts of aerosolised HAB toxins and air pollutants, especially in areas with freshwater experiencing heightened commercial activity. Comprehending the chemical transformations of these toxins is essential for improving HAB early warning systems and safeguarding public health in the context of climate change.

## Figures and Tables

**Table 1:** Research on cyanobacteria adhering to airborne particulate matter.

Technique	Results of Sampling	References
Illumina Miseq sequencing	Cyanobacteria adhere to PM_2.5_, accounting for 8.53% as the second most abundant genus of the microbial community	[31]
High-throughput sequencing and qPCR	The results show that in the bacterial community structure attached to PM_2.5_ during the winter heating period in Jinan City, cyanobacteria accounted for 15.95% of the third abundant phylum	[32]
High-throughput sequencing, PCR amplifications, and taxonomic classification	Abundant cyanobacteria are found in more than 80% of PM_10_ samples	[33]
High-throughput sequencing	Higher relative abundance of cyanobacteria was detected in PM_2.5_ samples	[34]
High-throughput sequencing	Higher abundance of cyanobacteria in some specific samples	[35]
High-throughput sequencing	Analysis of the bacterial community structure in TSP, PM_10_, and PM_2.5_ revealed an average relative abundance of 18% cyanobacteria	[36]
Algae culture	Cyanobacteria were the dominant, comprising 23 genera and accounting for 67.6% of the total algal genera recorded, followed by green algae (23.5%) and others	[37]

## Data Availability

The data presented in this study are available upon request from the corresponding author.

## References

[1] Ho J.C., Michalak A.M., Pahlevan N. (2019). Widespread global increase in intense lake phytoplankton blooms since the 1980s. Nature.

[2] Plaas H.E., Paerl H.W. (2021). Toxic Cyanobacteria: A Growing Threat to Water and Air Quality. Environ. Sci. Technol..

[3] Ariel C.T., Yehonatan S., Naama L. (2024). Aeromicrobiology: A global review of the cycling and relationships of bioaerosols with the atmosphere. Sci. Total. Environ..

[4] Dillon K.P., Correa F., Judon C., Sancelme M., Fennell D.E., Delort A.-M., Amato P., Stams A.J.M. (2020). Cyanobacteria and Algae in Clouds and Rain in the Area of puy de Dôme, Central France. Appl. Environ. Microbiol..

[5] May N.W., Axson J.L., Watson A., Pratt K.A., Ault A.P. (2016). Lake spray aerosol generation: A method for producing representative particles from freshwater wave breaking. Atmos. Meas. Tech..

[6] Gobler C.J., Doherty O.M., Hattenrath-Lehmann T.K., Griffith A.W., Kang Y., Litaker R.W. (2017). Ocean warming since 1982 has expanded the niche of toxic algal blooms in the North Atlantic and North Pacific oceans. Proc. Natl. Acad. Sci. USA.

[7] Lim C.C., Yoon J., Reynolds K., Gerald L.B., Ault A.P., Heo S., Bell M.L. (2023). Harmful algal bloom aerosols and human health. eBioMedicine.

[8] Jia J., Chen Q., Lauridsen T. (2016). A Systematic Investigation into the Environmental Fate of Microcystins and The Potential Risk: Study in Lake Taihu. Toxins.

[9] Melaram R., Newton A.R., Lee A., Herber S., El-Khouri A., Chafin J. (2024). A review of microcystin and nodularin toxins derived from freshwater cyanobacterial harmful algal blooms and their impact on human health. Toxicol. Environ. Health Sci..

[10] Picardo M., Filatova D., Nuñez O., Farré M. (2019). Recent advances in the detection of natural toxins in freshwater environments. TrAC Trends Anal. Chem..

[11] Olson N.E., Cooke M.E., Shi J.H., Birbeck J.A., Westrick J.A., Ault A.P. (2020). Harmful Algal Bloom Toxins in Aerosol Generated from Inland Lake Water. Environ. Sci. Technol..

[12] Sharma N.K., Rai A.K. (2008). Allergenicity of airborne cyanobacteria Phormidium fragile and Nostoc muscorum. Ecotoxicol. Environ. Saf..

[13] Ault A.P., Moffet R.C., Baltrusaitis J., Collins D.B., Ruppel M.J., Cuadra-Rodriguez L.A., Zhao D., Guasco T.L., Ebben C.J., Geiger F.M. (2013). Size-Dependent Changes in Sea Spray Aerosol Composition and Properties with Different Seawater Conditions. Environ. Sci. Technol..

[14] Patterson J.P., Collins D.B., Michaud J.M., Axson J.L., Sultana C.M., Moser T., Dommer A.C., Conner J., Grassian V.H., Stokes M.D. (2016). Sea Spray Aerosol Structure and Composition Using Cryogenic Transmission Electron Microscopy. ACS Cent. Sci..

[15] Slade J.H., Vanreken T.M., Mwaniki G.R., Bertman S., Stirm B., Shepson P.B. (2010). Aerosol production from the surface of the Great Lakes. Geophys. Res. Lett..

[16] May N.W., Gunsch M.J., Olson N.E., Bondy A.L., Kirpes R.M., Bertman S.B., China S., Laskin A., Hopke P.K., Ault A.P. (2018). Unexpected Contributions of Sea Spray and Lake Spray Aerosol to Inland Particulate Matter. Environ. Sci. Technol. Lett..

[17] Rogers M., Stanley R. (2023). Airborne Algae: A Rising Public Health Risk. Environ. Sci. Technol..

[18] Wood S.A., Dietrich D.R. (2011). Quantitative assessment of aerosolized cyanobacterial toxins at two New Zealand lakes. J. Environ. Monit..

[19] May N.W., Olson N.E., Panas M., Axson J.L., Tirella P.S., Kirpes R.M., Craig R.L., Gunsch M.J., China S., Laskin A. (2017). Aerosol Emissions from Great Lakes Harmful Algal Blooms. Environ. Sci. Technol..

[20] Nie C., Qiu Y., Pei T., Qin Y. (2024). Specific Sources Exert Influence on the Community Structures of Bioaerosols. Aerobiology.

[21] Fröhlich-Nowoisky J., Kampf C.J., Weber B., Huffman J.A., Pöhlker C., Andreae M.O., Lang-Yona N., Burrows S.M., Gunthe S.S., Elbert W. (2016). Bioaerosols in the earth system: Climate, health, and ecosystem interactions. Atmos. Res..

[22] Lewandowska A.U., Śliwińska-Wilczewska S., Woźniczka D. (2017). Identification of cyanobacteria and microalgae in aerosols of various sizes in the air over the Southern Baltic Sea. Mar. Pollut. Bull..

[23] Sahu N., Tangutur A.D. (2015). Airborne algae: Overview of the current status and its implications on the environment. Aerobiologia.

[24] Vejerano E.P., Ahn J., Scott G.I. (2024). Aerosolized algal bloom toxins are not inert. Environ. Sci. Atmos..

[25] Wiśniewska K., Lewandowska A.U., Śliwińska-Wilczewska S. (2019). The importance of cyanobacteria and microalgae present in aerosols to human health and the environment—Review study. Environ. Int..

[26] Harb C., Foroutan H. (2022). Experimental development of a lake spray source function and its model implementation for Great Lakes surface emissions. Atmos. Chem. Phys..

[27] Tormo R., Recio D., Silva I., Muñoz A.F. (2001). A quantitative investigation of airborne algae and lichen soredia obtained from pollen traps in south-west Spain. Eur. J. Phycol..

[28] Huang Z., Yu X., Liu Q., Maki T., Alam K., Wang Y., Xue F., Tang S., Du P., Dong Q. (2024). Bioaerosols in the atmosphere: A comprehensive review on detection methods, concentration and influencing factors. Sci. Total Environ..

[29] Shen F., Yao M. (2023). Bioaerosol nexus of air quality, climate system and human health. Natl. Sci. Open.

[30] Xie W., Li Y., Bai W., Hou J., Ma T., Zeng X., Zhang L., An T. (2020). The source and transport of bioaerosols in the air: A review. Front. Environ. Sci. Eng..

[31] Xu C., Chen J., Wang Z., Chen H., Feng H., Wang L., Xie Y., Wang Z., Ye X., Kan H. (2021). Diverse bacterial populations of PM2.5 in urban and suburb Shanghai, China. Front. Environ. Sci. Eng..

[32] Wei M., Liu H., Chen J., Xu C., Li J., Xu P., Sun Z. (2020). Effects of aerosol pollution on PM2.5-associated bacteria in typical inland and coastal cities of northern China during the winter heating season. Environ. Pollut..

[33] Romano S., Becagli S., Lucarelli F., Rispoli G., Perrone M.R. (2020). Airborne bacteria structure and chemical composition relationships in winter and spring PM10 samples over southeastern Italy. Sci. Total Environ..

[34] Hu Z., Liu H., Zhang H., Zhang X., Zhou M., Lou L., Zheng P., Xi C., Hu B. (2020). Temporal discrepancy of airborne total bacteria and pathogenic bacteria between day and night. Environ. Res..

[35] Yang J., Seo J., Jee Y., Kim Y., Sohn J. (2023). Composition Analysis of Airborne Microbiota in Outdoor and Indoor Based on Dust Separated by Micro-sized and Nano-sized. Aerosol Air Qual. Res..

[36] Liu H., Zhang X., Zhang H., Yao X., Zhou M., Wang J., He Z., Zhang H., Lou L., Mao W. (2018). Effect of air pollution on the total bacteria and pathogenic bacteria in different sizes of particulate matter. Environ. Pollut..

[37] Sharma N., Singh S., Rai A. (2006). Diversity and seasonal variation of viable algal particles in the atmosphere of a subtropical city in India. Environ. Res..

[38] Schaeffer B.A., Urquhart E., Coffer M., Salls W., Stumpf R.P., Loftin K.A., Jeremy Werdell P. (2022). Satellites quantify the spatial extent of cyanobacterial blooms across the United States at multiple scales. Ecol. Indic..

[39] Stroming S., Robertson M., Mabee B., Kuwayama Y., Schaeffer B. (2020). Quantifying the Human Health Benefits of Using Satellite Information to Detect Cyanobacterial Harmful Algal Blooms Manage Recreational Advisories in, U.S. Lakes. GeoHealth.

[40] Zhao Y., Liu D., Wei X. (2020). Monitoring cyanobacterial harmful algal blooms at high spatiotemporal resolution by fusing Landsat and MODIS imagery. Environ. Adv..

[41] Yang J., Kim J., Jeon H., Lee J., Seo J. (2025). Integrated culture-based and metagenomic profiling of airborne and surface-deposited bacterial communities in residential environments. Environ. Pollut..

[42] Shi J.H., Birbeck J.A., Olson N.E., Parham R.L., Holen A.L., Ledsky I.R., Ramakrishna B.S., Bilyeu L., Jacquemin S.J., Schmale D.G. (2025). Detection of Aerosolized Anabaenopeptins from Cyanobacterial Harmful Algal Blooms in Atmospheric Particulate Matter. ACS Earth Space Chem..

[43] Yu S., Zhou X., Hu P., Chen H., Shen F., Yu C., Meng H., Zhang Y., Wu Y. (2022). Inhalable particle-bound marine biotoxins in a coastal atmosphere: Concentration levels, influencing factors and health risks. J. Hazard. Mater..

[44] Després V.R., Huffman J.A., Burrows S.M., Hoose C., Safatov A.S., Buryak G., Fröhlich-Nowoisky J., Elbert W., Andreae M.O., Pöschl U. (2012). Primary biological aerosol particles in the atmosphere: A review. Tellus B Chem. Phys. Meteorol..

[45] Schlichting H.E. (1969). The Importance Of Airborne Algae and Protozoa. J. Air Pollut. Control Assoc..

[46] Heise H.A. (1949). Symptoms of hay fever caused by algae. J. Allergy.

[47] Hu J., Liu J., Zhu Y., Diaz-Perez Z., Sheridan M., Royer H., Leibensperger R., Maizel D., Brand L., Popendorf K.J. (2020). Exposure to Aerosolized Algal Toxins in South Florida Increases Short- and Long-Term Health Risk in Drosophila Model of Aging. Toxins.

[48] Sun Y., Guo Y., Xu C., Liu Y., Zhao X., Liu Q., Jeppesen E., Wang H., Xie P. (2023). Will “Air Eutrophication” Increase the Risk of Ecological Threat to Public Health?. Environ. Sci. Technol..

[49] Breidenbach J.D., French B.W., Gordon T.T., Kleinhenz A.L., Khalaf F.K., Willey J.C., Hammersley J.R., Mark Wooten R., Crawford E.L., Modyanov N.N. (2022). Microcystin-LR aerosol induces inflammatory responses in healthy human primary airway epithelium. Environ. Int..

[50] Schaefer A.M., Yrastorza L., Stockley N., Harvey K., Harris N., Grady R., Sullivan J., McFarland M., Reif J.S. (2020). Exposure to microcystin among coastal residents during a cyanobacteria bloom in Florida. Harmful Algae.

[51] Stewart I., Webb P.M., Schluter P.J., Fleming L.E., Burns J.W., Gantar M., Backer L.C., Shaw G.R. (2006). Epidemiology of recreational exposure to freshwater cyanobacteria—An international prospective cohort study. BMC Public Health.

[52] Geng X., Nie C., Wang L., Li L., Li D., Nishino A., Chen J. (2023). ITS and 16S rRNA Gene Revealed Multitudinous Microbial Contaminations of Residential Air Conditioning Filters in Megacity Shanghai, China. Environ. Health.

[53] Nie C., Geng X., Zhang R., Wang L., Li L., Chen J. (2023). Abundant Cyanobacteria in Autumn Adhering to the Heating, Ventilation, and Air-Conditioning (HVAC) in Shanghai. Microorganisms.

[54] Nie C., Geng X., Ouyang H., Wang L., Li Z., Wang M., Sun X., Wu Y., Qin Y., Xu Y. (2023). Abundant bacteria and fungi attached to airborne particulates in vegetable plastic greenhouses. Sci. Total Environ..

